# Subjective Cognitive Decline and Nighttime Sleep Alterations, a Longitudinal Analysis

**DOI:** 10.3389/fnagi.2019.00142

**Published:** 2019-07-02

**Authors:** Giovanna Bubbico, Angelo Di Iorio, Mariella Lauriola, Gianna Sepede, Simone Salice, Eleonora Spina, Giacomo Brondi, Roberto Esposito, Mauro Gianni Perrucci, Armando Tartaro

**Affiliations:** ^1^Department of Neuroscience, Imaging and Clinical Sciences, “G. d’Annunzio” University of Chieti-Pescara, Chieti, Italy; ^2^Institute for Advanced Biomedical Technologies (ITAB), “G. d’Annunzio” University of Chieti-Pescara, Chieti, Italy; ^3^Department of Medicine and Science of Aging, “G. d’Annunzio” University of Chieti-Pescara, Chieti, Italy; ^4^Momentum for Mental Health, La Selva, Palo Alto, CA, United States; ^5^Department of Radiology, Azienda Ospedaliera Ospedali Riuniti Marche Nord, Pesaro, Italy; ^6^Radiology Units, Popoli Hospital, Popoli, Italy

**Keywords:** sleep, aging, prevention, dementia, subjective cognitive decline

## Abstract

**Objective**: The aim of this study was to analyze quantitative sleep changes and their implication on subjective cognitive decline (SCD). Objective sleep patterns were investigated by an actigraph and recorded at the baseline and 2-year after in order to examine specific sleep alterations in SCD.

**Background**: Sleep disorders are very common among average elderly adults and an altered sleep pattern is known to be a risk factor for future development of mild cognitive impairment (MCI) and dementia. Recent studies have shown how sleep is objectively altered in average senior adults with SCD, without any other significant change in cognition and behavior or brain structure. Considering that both SCD and disrupted sleep are risk factors for future MCI and dementia, with sleep only as a modifiable risk factor, further research is required to deeply investigate the interaction between sleep and SCD.

**Methods**: Among 70 community-dwelling elderly individuals who had been enrolled at baseline, 35 (64.6 ± 5.6 years, 15 M/20 F) underwent a complete neuropsychological battery and 1-week wrist actigraphy recording 2 years later during the follow-up stage. Individuals were divided into two groups according to their SCD Questionnaire (SCD-Q) score. Sleep hours, sleep efficiency and onset latency, napping and time awake after sleep onset (WASO) were collected. All individuals underwent structural magnetic resonance imaging (MRI) examination to exclude brain disorders. Data collection was performed at baseline and after 2 years at the follow-up phase.

**Results**: A significantly different night sleep time between the two groups was observed: SCD showed a lower total sleep time (TST) than non-SCD subjects. Moreover, a total time spent in bed (TIB) was significantly lower in SCD subjects over 2 years of observation.

**Conclusions**: Objective changes over time of the sleep pattern, specifically TIB and TST, are present in SCD individuals. The results of the study show that sleep alterations are common in SCD and underline the clinical importance of screening in order to assess sleep alterations as well as improve sleep in average adults with SCD complaints.

## Introduction

Sleep disturbances are frequent symptoms in adults and are associated with various factors, including health-related quality of life, chronic diseases and mental health (Chen et al., 2014), moreover sleep disorders are also associated with age-related cognitive decline (Bliwise, 2004; Bliwise et al., 2005; Bishop et al., 2010; Mander et al., 2017; Génier Marchand et al., 2018). However, nowadays, it is still debated whether sleep problems may themselves trigger or exacerbate chronic diseases in adults (Koyanagi et al., 2014). A large amount of research describes how sleep disorders have a significant effect on both patients with Alzheimer’s Disease (AD) and their caregivers, because of the most worrisome and manifest symptoms during the progression of dementia (Sprecher et al., 2017; Brzecka et al., 2018; Génier Marchand et al., 2018). Emerging evidence suggests the existence of an interaction between the deteriorated sleep-wake cycle and dementia (Hita-Yañez et al., 2013; van Oostrom et al., 2018). Recent studies suggest that sleep disorders anticipate by years the clinical onset of dementia (Iranzo et al., 2006; Fulda, 2011). Sleep alterations in preclinical stages of AD remain still underrecognized by many healthcare professionals. One of the reasons might be the common belief that sleep problems are normal signs of aging regardless of the patient’s pre-dementia condition. A simple short sleep interview may help in order to investigate differences between memory decline caused by normal aging or other dementia pathophysiology and therefore a more complete sleep test can be a useful tool to monitor pathological aging.

Subjective cognitive decline (SCD) refers to individuals who perceive a decline in memory and/or other cognitive abilities related to their previous level of performance, in the absence of objective neuropsychological deficit (Fernández-Blázquez et al., 2016; Eckerström et al., 2017; Ávila-Villanueva and Fernández-Blázquez, 2017). A large number of studies show that elderly individuals with SCD have an increased likelihood of biomarker anomalies which are consistent with an increased risk for future pathologic cognitive decline and AD. Recently SCD has been proposed as a pre-mild cognitive impairment (MCI) stage (Jessen et al., 2014; Jessen, 2014); therefore many investigations focus on SCD, with the aim of finding a biomarker linking this pre-dementigenous stage with dementia.

What sleep disorders and degenerative cognitive decline have in common still needs to be investigated. Researches show that sleep disorders, such as fragmented sleep or reduced total sleep time (TST) or increased night-time wake are very common in patients with MCI and AD (Jelicic et al., 2002; Ju et al., 2013, 2014; da Silva, 2015; Dubois et al., 2016). Few studies show that sleep is altered in the SCD individuals (Lauriola et al., 2016; Nakakubo et al., 2018; Tsapanou et al., 2018).

Moreover, the risk of future cognitive decline increases in elderly suffering from sleep complaints (Spira et al., 2014). Exploring the link between sleep alterations and the onset of cognitive problems in the elderly could allow targeting educational and effective clinical programs to improve sleep quality of patients with SCD, by enhancing the quality of life of this population and possibly preventing the onset of objective cognitive problems. Furthermore, key factors affecting sleep efficacy in subjects with SCD would lead to better strategies in terms of costs and benefits in healthcare systems and particularly actigraphy techniques can be a useful and affordable tool to this purpose. In this framework, the present study has been included with the aim of evaluating the relationship between the measurement of sleep pattern in SCD and non-SCD subjects. The hypothesis was that individuals with SCD would longitudinally show a more altered sleep pattern in the analysis of actigraphy sleep variables than their counterpart.

## Materials and Methods

**Figure 1:**
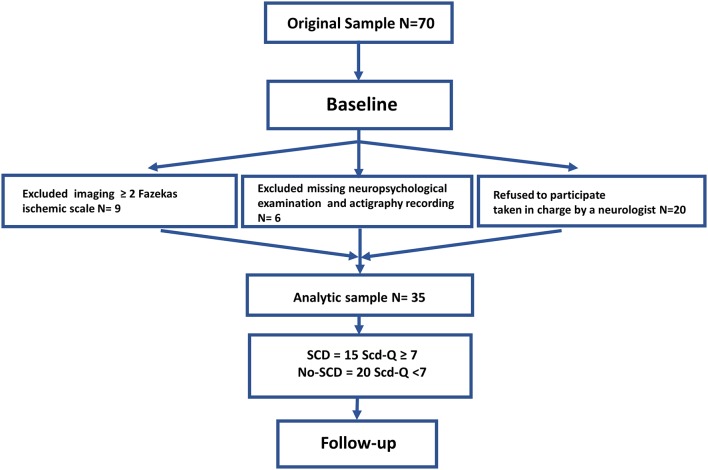
Flowchart describing sample selection.

Participants were recruited through advertisements in the Abruzzo region in Italy. Seventy subjects were enrolled at baseline, 35 subjects (20 females, 15 males; mean age: 64.6 ± 5.6 years) participated in the follow-up stage 2 years later. Nine subjects were excluded due to periventricular and deep white matter (WM) damage, 20 subjects refused to participate because they were under the supervision of a neurologist not involved in the study, and six were missing neuropsychological or actigraphy data (see Figure 1). Experimental procedures were previously approved by the Ethical Committee for Human Research at the G. d’Annunzio University of Chieti-Pescara, Italy, and all participants signed an informed consent.

### Design, Setting, and Participants

The study was a longitudinal cohort analysis. All participants underwent a medical and neuropsychological assessment, an apnea screening, actigraphy sleep recording and magnetic resonance imaging (MRI) scan of the brain, at baseline and 2 years later (follow-up stage). ApneaLink™ device (ResMed Corporation, Poway, Calif) was employed for a screening tool for sleep apnea (Erman et al., 2007). Subjects self-reporting any sleep disorders (e.g., breathing-related sleep disorders, leg movement disorders) were excluded. Self-reported unmoderated alcohol intake (more than 12 grams per day) was an exclusion criterion.

Other exclusion criteria, which were confirmed by bed partners or caregivers, were also: psychiatric disorders, a history of neurological conditions, a major medical illness (chronic renal, hepatic, pulmonary, or endocrine), the use of medication affecting the sleep-wake cycle (benzodiazepines, tricyclic and/or serotonin reuptake inhibitors, antipsychotics), the presence of depression symptoms [assessed with the abbreviated version of the Geriatric Depression Scale (GDS), using five as a cut-off score], breathing issues during sleep and unusual sleep schedules (i.e., shift work).

Overnight actigraphy recordings were obtained from 35 community-dwelling elderly, divided into two groups: SCD and non-SCD, according to the classification of the SCD Questionnaire (SCD-Q).

All participants underwent 3 Tesla Philips Achieva 3T Scanner (Philips Medical Systems, Best, Netherlands) examination to rule out cerebral infarction, brain tumor, hippocampal sclerosis, vascular malformations. 3D FLAIR volume isotropic turbo spin-echo acquisition (VISTA) imaging was used to assess WM hyperintensity (WMH) and microbleedings. Those participants who showed periventricular and/or deep WM damage, coming from scorings ≥2 on the Fazekas ischemic scale, and/or microbleedings were not included in the study.

Sleep parameters had been registered for the duration of 1 week. Subjects performed a complete neuropsychological evaluation together with self-assessment tests for anxiety (State-Trait Anxiety Inventory, STAI) and depression (GDS; Marc et al., 2008; Julian, 2011). The details of the baseline enrollment and analysis have already been published (Lauriola et al., 2017).

### Demographic Characteristics

The categorization of SCD was based on consensus criteria: people with subjective cognitive complaints and a score ≥7 on the SCD questionnaire. Participants were assigned to the SCD or non-SCD group according to the SCD research criteria that were: normal cognition on standardized cognitive battery which was accompanied by a subjective decline in cognitive capacity in comparison with a previously cognitive condition. Moreover, these were not associated with another medical/psychiatric condition or an acute event, together with a total score on the SCD-Q (SCD-Q score ≥7 was classified as SCD; SCD-Q scores <7 as no-SCD; Rami et al., 2014).

The SCD-Q is a validated questionnaire that assesses the presence of a subjective cognitive decay in abilities such as memory, attention, language or executive functions. This scale is made up of two parts: MyCog is filled by the subject, TheirCog by the caregiver. Both parts have 24 identical dichotomous questions (yes/no), that evaluate decline for memory performances, language and executive functions in the last 2 years of daily life. The SCD-Q score for MyCog and TheirCog ranges from 0 to 24, with higher scores associated with greater perceived cognitive changes (cut to be classified as SCD = 7). In the present work, the MyCog part was used.

Fifteen (6 females) participants met SCD criteria while 20 were included as Non-SCD (14 females) for this longitudinal study.

### Actigraphic Sleep

Sleep/wake patterns were objectively measured using actigraphy (Cole-Kripke algorithm; Tilmanne et al., 2009) which is a reliable and non-invasive technique based on individuals’ motor activity. Actigraphic data were registered for each participant using ActiSleep-BT monitors (Acti-Graph, Pensacola, FL, USA). Participants wore the device for at least seven consecutive days on their non-dominant wrist. Data were sampled at 60 Hz (1-min epoch) and analyzed offline using ActiLife software (ActiGraph).

The following parameters were calculated with Actilife Cole-Kripke algorithm: total time in bed and total sleep time (TIB, minutes; TST, minutes), sleep onset latency (SOL, minutes), wake after sleep onset (WASO, minutes), total number of awakenings, average length of the awakenings (minutes), awakening index (number of awakening per hour of sleep calculated as total number of awakenings/TST × 60) and sleep efficiency (SE, TST/TIB × 100). An identical protocol was repeated after 2 years.

### Statistical Analyses

All analyses were conducted using SAS software 9.3 release (SAS Institute Inc., Cary, NC, USA). All continuous variables were first evaluated before analysis to verify normality distribution (Proc Capability) and then transformed if necessary. For simplicity also for non-normal variables, data were calculated as the average of all the variable values ± the standard deviation, whereas statistical tests were done using transformed variables. Differences among groups (according to SCD-score classification) were evaluated by the generalized linear model (PROC GLM) for continuous variables. Categorical or ordinal variables were listed as numbers and percentages, differences between groups were evaluated with chi-square test and, were necessary, Fisher correction was applied (PROC Freq).

The longitudinal variation of sleep parameters was evaluated through Mixed Models (PROC Mixed; Singer, 1998).

In model A, the unconditional means model, the variation of sleep parameters in the entire enrolled population was evaluated. Model B, the unconditional growth model, included only the time effect in the analysis; in model C, the interaction model, the interaction between time and SCD-groups was considered. Finally, model D was the same model C, adjusted for age, sex and cognitive status.

Mixed Models Fit was evaluated through the Akaike Information Criterion (AIC) and Bayesian Information Criterion (BIC). In comparing the scores among the different models, the lowest scores provided more information about the analysis.

## Results

### Demographic and Cognitive Profile

Thirty-five subjects were enrolled for the follow-up study, 15 subjects for the SCD group (9 M/6 F; AGE 66.53–4.76; EDU 10.8, 3.14; SCD-Q 9.66–2.6), and 20 subjects (14 F/6 M), for the non-SCD group (AGE 63.15–5.91; EDU 10.7–3.74; SCD-Q 3.7–1.6). Both groups showed similar demographic profiles: age was comparable in the two groups (*p* = 0.41), and SCD participants had higher SCD-Q than their counterpart at the baseline (*p* = 0.007).

### Sleep Pattern

**Figure 2:**
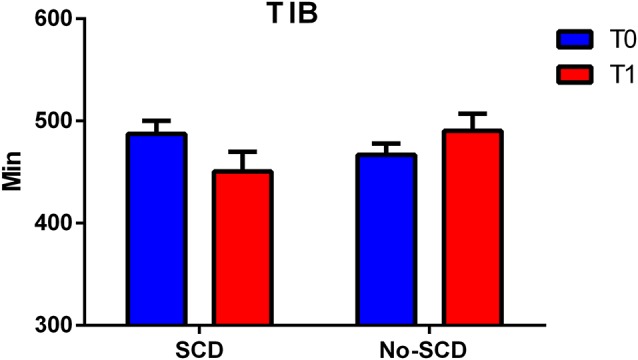
Figure shows differences between total minutes spent in bed (TIB) in subjective cognitive decline (SCD) and Non-SCD subjects at 2-year follow-up phase (TIB between group *p* = 0.02).

**Figure 3:**
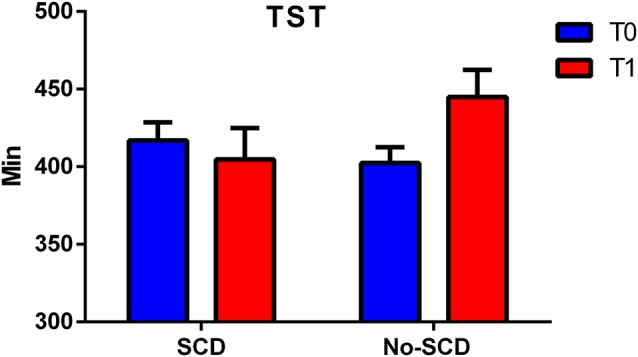
Figure shows differences between total sleep time (TST) in SCD and Non-SCD subjects at 2-year follow-up stage (sleep time between group *p* = 0.03).

The differences between the various sleep parameters (actigraphy) at the baseline or follow-up stages (supplementary information, Supplementary Material Appendix 1) were not significant, however when considering the interaction between the positivity to the SCD-q score and the study time, at both the baseline and follow-up stages, two sleep quality parameters resulted significantly different, more specifically the time spent in bed (TIB *p* = 0.03; see Figure 2); and the total sleep time (TST *p* = 0.02; see Figure 3).

Results of the mixed model analysis are shown in Tables s 1, 2. In model A, the unconditional means model was reported. This analysis established how much time was spent in bed by the entire enrolled population and how it varied over time. From this model, the intraclass correlation coefficient (ICC) was computed and the results for the TIB was 29.5% and for TST was 23.6%; this means that a quarter in the total variation of TIB and TST was attributable to differences among enrolled subjects. In model B, the effect of time was evaluated, which was not statistically significant both for TIB and TST. Furthermore, 1% of the variation for both the parameters was linked to time. In model C, the interactions between time and SCD-positive subjects were reported. According to the previous hypothesis, subjects without SCD showed in the initial stage a TIB of 490.30 ± 16.72 min/week, and a TST of 444.89 ± 17.45 min/week.

**Table 1:** Mixed model, analysis of variation of Total Time Spent in Bed (TIB) according to subjective cognitive decline-questionnaire (SCD-Q) classification.

			Model A Unconditional Means Model	Model B Unconditional Growth Model	Model C Interaction	Model D Interaction adjusted
Initial status	Intercept	γ_00_	475.10 ± 7.71	473.29 ± 13.07	490.30 ± 16.72	485.37 ± 151.17
	SCD-questionnaire	γ_01_			−39.70 ± 25.54	−39.58 ± 25.75
Rate of change	Time	γ_10_		2.40 ± 14.01	−23.40 ± 17.30	−23.57 ± 18.02
	Time*SCD	γ_11_			60.20 ± 26.42	60.2110 ± 26.42
	Initial status	$\delta_{0}^{2}$	2,501.96 ± 598.15	2,501.60 ± 598.00	2,398.43 ± 573.33	2,402.74 ± 598.52
	In rate of change	$\delta_{1}^{2}$	5,978.01 ± 1,429.29	5,974.74 ± 1,428.24	5,588.94 ± 1,336.01	5,573.85 ± 1,432.77
	Covariance	$\delta_{1}^{0}$	800.84 ± 667.64	801.92 ± 667.39	1,001.43 ± 641.59	996.73 ± 661.49
		$R_{0}^{2}$			0.041	0.040
		$R_{1}^{2}$			0.065	0.070
		AIC	783	781	777	776
		BIC	789	788	779	795

**Table 2:** Mixed model, analysis of variation of Total Sleep Time (TST) according to SCD-questionnaire classification.

			Model A Unconditional Means Model	Model B Unconditional Growth Model	Model C Interaction	Model D Interaction adjusted
Initial status	Intercept	γ_00_	410.97 ± 7.55	427.69 ± 13.61	444.89 ± 17.4565	316.06 ± 141.74
	SCD-questionnaire	γ_01_			−40.12 ± 26.66	−37.08 ± 25.96
Rate of change	Time	γ_10_		−19.08 ± 12.92	−42.46 ± 15.99	−46.81 ± 16.65
	Time*SCD	γ_11_			54.55 ± 24.42	54.40 ± 24.37
	Initial status	$\delta_{0}^{2}$	2,091.82 ± 501.35	2,086.42 ± 498.75	2,035.46 ± 486.57	2,057.87 ± 498.43
	In rate of change	$\delta_{1}^{2}$	6,768.73 ± 1,638.87	6,488.90 ± 1,551.14	6,094.62 ± 1,456.89	5,681.64 ± 1,437.32
	Covariance	$\delta_{1}^{0}$	1,326.59 ± 683.61	1,365.94 ± 663.42	1,507.69 ± 647.60	1,324.84 ± 649.24
		$R_{0}^{2}$			0.024	0.019
		$R_{1}^{2}$			0.001	0.124
		AIC	778	769	762	760
		BIC	784	778	771	768

Subjects resulting positive in SCD spent less time in bed than their counterpart (−39.70 ± 25.54, Table 2, model C). Finally, on average, subjects without-SCD spent more time in bed and more time sleeping (−40.12 ± 26.66, Table 1, model C), which was a statistically significant value.

Model D also accounted for the effect of age, sex, and Mini Mental State Examination (MMSE). By analyzing the AIC and BIC values (statistical fit of the model), the models did not improve. For this reason, Model C was the simplest and most parsimonious model to be considered.

## Discussion

The aim of this study was to examine sleep alterations in community-dwelling individuals with SCD at the baseline phase and at follow-up 2 years later. For this purpose, 70 elderly subjects were included at baseline stage in the study. Of these, 35 subjects participate in the follow-up phase and underwent a complete study protocol, including a neuropsychological examination, brain MRI scan, to exclude any neuropathological conditions, and 1-week sleep actigraphy recording.

In the present study, it was evaluated if objective sleep actigraphy recording differed between SCD and non-SCD subjects, by analyzing meaningful group differences in sleep pattern at the baseline stage and after 2 years. The results provided compelling evidence of TST differences between groups. Sleep alteration remained significantly different even considering states of anxiety and depression as a covariate in the statistical model which was used. At the follow-up stage 2 years later, the SCD group showed a significant reduction in sleep duration whereas the non-SCD group showed an increase in sleep duration.

Sleep alterations are prevalent among older adults who are at risk of developing dementia (Palmer et al., 2018; Lucey et al., 2019). So far, there have been few studies that have analyzed the objective quality of sleep in these individuals, most of them have considered subjective parameters (Cavuoto et al., 2015; Landry et al., 2015; Smagula et al., 2016; Lysen et al., 2018; Tsapanou et al., 2018).

Sleep disorders are common in MCI (Palmer et al., 2018), for example, waking up at night is a specific symptom. Showing that overall poor sleep duration is present in SCD subjects, the present study takes into consideration a population that might be in a pre-clinical state and prior to the condition of MCI, notably in a phase where treatments could play a role in the prevention or delay of the disease. Moreover, poor sleep quality has been associated with increased cortical atrophy in community-dwelling adults (Sexton et al., 2014). Therefore, future research will be necessary to explore this aspect also in the SCD population. In line with the previous studies of Lauriola et al. (2016, 2017), objective sleep was altered in SCD participants, who showed at the baseline phase, increased nighttime wakefulness and reduced sleep efficiency.

There are several studies underling the connection between the risk of developing AD and the quality of sleep (Tworoger et al., 2006; Barnard et al., 2014; Brzecka et al., 2018). Similar data were observed in a Spanish study of 3,212 people aged ≥60 years, here authors found no correlation between reduced sleep time (<7 h; according to self-reported data) and cognitive disorders which are determined by MMSE questionnaire (Faubel et al., 2009).

The results of the present analysis may support the positive correlation between sleep impairment and SCD as well as the association between objective sleep-wake alterations and risk of cognitive decline in a cohort of frail elderly individuals. Here in the study participants were targeted as a population at risk and their pre-existing condition may have contributed to the sleep deterioration. The actigraphy registration in this study allowed a deep objective profile of the sleep trend in the two observed populations. Other studies conducted among older adults have found a correlation between the worst cognitive abilities and the too short or too long duration of sleep. In particular, Ramos et al. (2013) reported an association of inverted U-forms with worse scores in neurocognitive functions and a long sleep duration (Ohayon and Vecchierini, 2005; Potvin et al., 2012; Benito-León et al., 2013; Ramos et al., 2013; Devore et al., 2014). With our limited group of subjects with SCD, we could not find any significant correlation with cognitive abilities, and therefore longitudinal studies using objective sleep measurements are needed to analyze this problem.

These different results may be caused by dissimilar follow-up measures and populations. Studies that observe sleep disorders in the MCI and AD population are numerous, whereas the number of studies that observe objective sleep patterns and cognitive performance in subjects with SCD is lacking. In recent studies of participants with subjective memory disorders, Mander (2013) reported a worse overall quality of sleep which was significantly associated with perceived subjective decline. In line with this result, the present study supports these findings and highlights how the quality of sleep worsens in SCD over time (2 years).

Tsapanou et al. (2018) found more sleep problems associated with an increased amount of SCD level and sleep problems, in this case, were associated in their study with subjective memory issues, particularly naming and calculations in two large cohorts of cognitively healthy older adults. They found in an unadjusted multinomial regression model, that more subjective sleep problems were associated with more total SCD (Tsapanou et al., 2018).

Unlike their study, the present analysis has a small sample of subjects because of the study design, that is participants taking medication such as narcotics, hypnotics, antipsychotics and anticholinergics were excluded whereas they can modify and influence cognitive performances or sleep, as well as the circadian rhythm (Proctor and Bianchi, 2012; Boucart et al., 2015a,b; De Berardis et al., 2015). The present analysis included a small sample of subjects; therefore, findings need to be confirmed in a larger analysis.

Furthermore, unlike their study, the present work provides an objective measure of sleep recorded through actigraphy, with a 1-week recording method, furthermore, the current research procedure gives a comparison of data in a longitudinal manner.

Nocturnal sleep reduction may also be present even in the absence of dementia conditions in healthy elderly subjects (Monjan, 2010; Scullin and Bliwise, 2015; Mander et al., 2017). Therefore, an alternative explanation of our results could be that a reduction in sleep may explain by itself a condition of SCD. In this case, an intervention on sleep could have a positive effect on the improvement of sleep quality but also on reducing or eliminating subjective cognitive complaints.

## Conclusion

In conclusion, although sleep disorders are a prevalent component in the geriatric population and in patients with dementia, they remain under-diagnosed. In the present study, simple and affordable nocturnal recordings were employed to determine sleep alterations in individuals with SCD.

Such observations of sleep-wake rhythm can give important information about the onset of cognitive deterioration, even years before the clinical onset of dementia. Sleep deterioration should have an outstanding place in the study of the prodromal stages of dementia and may accompany cognitive aging regardless of incipient neurodegeneration. The results of the present study may also have important implications for the early diagnosis of possible pathological aging and could help in the development of new therapeutic strategies dedicated to improving sleep in elderly subjects with SCD. As sleep is significantly impaired in patients with SCD, sleep alterations might be used as a surrogate marker of the preclinical stage even in SCD. Altogether, these findings can help in the development of new therapeutic strategies devoted to improving sleep in the elderly population with a risk of developing dementia (Galtier et al., 2019).

To sum up, the results of the present study showed that objective sleep pattern in SCD differs from non-SCD healthy elderly individuals and this difference increases over time. In particular, variables as TST and TIB decreased after a 2-year actigraphy observation.

Further longitudinal studies are needed to validate the altered sleep pattern as a potential and early biomarker of pathological aging.

## Ethics Statement

This study was performed in accordance with the recommendations of the Declaration of Helsinki. All subjects signed written informed consent. The protocol was approved by the Ethics Committee of G. d’Annunzio University of Chieti, Italy.

## Author Contributions

ML, AT, and GBu conceived the idea and designed the research for the follow-up phase. GBu and ML supervised the experiment. ES and GBr exported the data. AT, MP, GBu, RE, ADI, ML, and GS reviewed the article. ADI and GS ran the statistical analysis. AT and MP supervised the research and AT reviewed the article for intellectual content. AT, SS, and RE did the neuroradiological examinations. GBu,~ADI, and~AT interpreted the results. GBu~and~AT wrote the first version of the manuscript. All authors approved the final version.

## Conflict of Interest Statement

The authors declare that the research was conducted in the absence of any commercial or financial relationships that could be construed as a potential conflict of interest.
