# Supplementary material for: Subjective Cognitive Decline and Nighttime Sleep Alterations, a Longitudinal Analysis
Source: Front Aging Neurosci. 2019 Jul 2;11:142. doi: 10.3389/fnagi.2019.00142 (PMC6614445; doi:10.3389/fnagi.2019.00142)
Supplement: Supplementary file 1 [file Table_1.docx]

**Appendix Table 1: Mixed Linear Models analyzing difference among SCD-groups according to time.**

|  |  |  |  |  |  |  | Time*group |
| --- | --- | --- | --- | --- | --- | --- | --- |
|  | T0 G0 | T0G1 | p* | T1G0 | T1G1 | p** | P ^§^ |
| LATENCY | 7.23±4.85 | 6.00±4.59 | 0.45 | 0.89±0.48 | 1.14±0.69 | 0.21 | 0.19 |
| TOTAL_COUNTS | 50409.45±27144.89 | 59962.93±30699.89 | 0.38 | 35104.56±6882.37 | 29496.52±10416.39 | 0.06 | 0.11 |
| EFFICIENCY | 86.63±5.66 | 85.67±4.37 | 0.59 | 90.01±3.66 | 89.64±3.90 | 0.78 | 0.68 |
| TIB | 466.89±41.01 | 487.42±60.94 | 0.24 | 490.30±88.29 | 450.61±58.24 | 0.14 | 0.02 |
| TST | 402.43±39.98 | 416.86±54.03 | 0.37 | 444.89±94.31 | 404.77±56.27 | 0.15 | 0.03 |
| WASO | 56.85±28.69 | 64.51±22.51 | 0.40 | 44.52±13.81 | 44.70±17.24 | 0.97 | 0.60 |
| NAW | 15.08±5.46 | 16.40±3.41 | 0.42 | 11.97±4.41 | 13.04±3.55 | 0.45 | 0.80 |
| AAL | 3.80±1.07 | 4.18±1.15 | 0.32 | 4.00±1.24 | 3.53±0.99 | 0.23 | 0.10 |
| AWI | 2.28±0.91 | 2.40±0.55 | 0.68 | 1.82±0.81 | 2.03±0.74 | 0.43 | 0.63 |
| SE | 0.87±0.06 | 0.86±0.04 | 0.62 | 0.90±0.04 | 0.90±0.04 | 0.78 | 0.74 |

**Table 1 Appendix**: Actigraphy sleep variables in No-SCD and SCD. Mixed Linear Models analyzing difference among groups according to time. Results are expressed in mean and standard deviation.

*p-value = between groups at baseline;

**p-value = between groups at follow-up;

^§^ p-value for the interaction among Time*SCD-groups

Abbreviations: sleep onset latency (SOL, minutes), sleep efficiency, Total time in bed (TIB, minutes), total sleep time (TST, minutes), wake after sleep onset (WASO, minutes), NAW total number of awakenings, average length of the awakenings (minutes), awakening index (AWI, number of awakening per hour of sleep calculated as total number of awakenings/TST x 60) and sleep efficiency (SE, TST/TIB x 100).
